# Robot-assisted Simple Prostatectomy with Tunnel-Shaped Trigonization (RASP-TST) – A Novel Technique

**DOI:** 10.1590/S1677-5538.IBJU.2018.0611

**Published:** 2019-09-02

**Authors:** Marcos Tobias-Machado, Cristiano Linck Pazeto, Eliney Ferreira Faria, Breno Dauster, William Enrique Pertuz Genes, Ricardo Hissashi Nishimoto

**Affiliations:** 1 Departamento de Urologia, Faculdade de Medicina do ABC, Santo André, SP, Brasil;; 2 Departmento de Urologia Hospital do Câncer de Barretos, Barretos, SP, Brasil;; 3 Serviço de Urologia, Hospital São Rafael, Salvador, BA, Brasil;; 4 Departamento de Urologia Hospital Alberto Cavalcanti, Belo Horizonte, MG, Brasil

## Abstract

To describe a technical modification for robotic-assisted simple prostatectomy (RASP) using three-steps reconstruc¬tive technique to achieve a 360 trigonization of the bladder mucosa. Through five-trocars transperitoneal access, we perform a longitudinal incision of the bladder wall and prostate capsule. Our technique of RASP is very similar to the standard operative technique described during laparoscopic and robotic removal of adenoma, however, for reconstruction, we propose the Tunnel-Shaped Trigonization (TST). The first step is the advancement of a bladder mucosa flap until the posterior part of the prostatic urethra. The second step, a running suture between the advanced mucosa and the prostatic capsule is done bilaterally. At this point, the prostate capsule should be totally isolated from the rest of the urinary tract. Finally, the third step is closing both sides of the capsule and bladder mucosa anteriorly identical to a tunnel conformation. Hiding the prostatic capsule optimizes the patient recovery since hematuria is the most related factor for hospital stay length. This pilot-case has shown satisfactory results without the need for continuous bladder irrigation. The prostate volume in the TRUS was 130 cm3 and the preoperative International Prostate Symptom score was 24. He was discharged at second postoperative day and no late complications were detected. In conclusion, the TST-RASP seems to be a safe and feasible modification of the RASP. We hope that the application of the TST can lead us to lower rates of blood loss, transfusion and postoperative complications in comparison to the standard technique.

ARTICLE INFO

*Cristiano Linck Pazeto*

http://orcid.org/0000-0003-3662-6454

Available at: http://www.intbrazjurol.com.br/video-section/20180611_Tobias-Machado_et_al

Int Braz J Urol. 2019; 45 (Video #17): 858-858

